# One year outcomes of a mentoring scheme for female academics: a pilot study at the Institute of Psychiatry, King's College London

**DOI:** 10.1186/1472-6920-11-13

**Published:** 2011-04-07

**Authors:** Rina Dutta, Sarah L Hawkes, Elizabeth Kuipers, David Guest, Nicola T Fear, Amy C Iversen

**Affiliations:** 1 King's College London, Department of Psychosis Studies, Institute of Psychiatry, London, UK; 2 King's College London, Department of Psychological Medicine, Institute of Psychiatry, London, UK; 3 King's College London, Department of Management, London, UK; 4 King's College London, Academic Centre for Defence Mental Health, Institute of Psychiatry, London, UK; 5 King's College London, Department of Academic Psychiatry, Institute of Psychiatry, London, UK

## Abstract

**Background:**

The professional development of under-represented faculty may be enhanced by mentorship, but we understand very little about the mechanisms by which mentoring brings about change. Our study posed the research question, what are the mechanisms by which mentoring may support professional development in under-represented groups?

The study aims to: (i) to pilot a mentoring scheme for female academics; (ii) to compare various health-related and attitudinal measures in mentees at baseline, 6 months, and 1 year into the mentoring relationship and, (iii) to compare pre-mentoring expectations to outcomes at 6 months and 1 year follow-up for mentees and mentors.

**Methods:**

Female academic mentees were matched 1:1 or 2:1 with more senior academic mentors. Online surveys were conducted to compare health-related and attitudinal measures and expectations of mentoring at baseline with outcomes at 6 months and 1 year using paired t-tests and McNemar's test for matched cohort data.

**Results:**

N = 46 mentoring pairs, 44 (96%) mentees completed the pre-mentoring survey, 37 (80%) at 6 months and 30 (65%) at 1 year. Job-related well-being (anxiety-contentment), self-esteem and self-efficacy all improved significantly and work-family conflict diminished at 1 year. Highest expectations were career progression (39; 89%), increased confidence (38; 87%), development of networking skills (33; 75%), better time-management (29; 66%) and better work-life balance (28; 64%). For mentees, expectations at baseline were higher than perceived achievements at 6 months or 1 year follow-up.

For mentors (N = 39), 36 (92%) completed the pre-mentoring survey, 32 (82%) at 6 months and 28 (72%) at 1 year. Mentors' highest expectations were of satisfaction in seeing people progress (26; 69%), seeing junior staff develop and grow (19; 53%), helping solve problems (18; 50%), helping women advance their careers (18; 50%) and helping remove career obstacles (13; 36%). Overall, gains at 6 months and 1 year exceeded pre-mentoring expectations.

**Conclusions:**

This uncontrolled pilot study suggests that mentoring can improve aspects of job-related well-being, self-esteem and self-efficacy over 6 months, with further improvements seen after 1 year for female academics. Work-family conflict can also diminish. Despite these gains, mentees' prior expectations were shown to be unrealistically high, but mentors' expectations were exceeded.

## Background

In academic institutions women advance more slowly in rank than men, hold fewer leadership positions, receive lower salaries and obtain fewer research grants [1,2]. Although various factors may explain these differences [3], including greater conflict between work and family responsibilities for women, a lack of effective mentors is cited as one potential reason for the disparity [4].

There is some evidence from the USA that introducing mentoring schemes improves career trajectories for female academics [5,6] as well as under-represented minorities [7]. The recent 'Women in Academic Medicine' report from the British Medical Association strongly recommended that "mentoring for women staff should be mainstreamed and monitored" and that institutions should "establish mentoring schemes as an essential and valuable activity" with time for mentoring "recognised in job plans" in order to attract and retain females in academic career paths [8].

The existing evidence base for mentoring is limited [9]. Most studies to date have been small, cross-sectional studies collecting data relating to either mentees or mentors (not simultaneously) and relying on questionnaires which have not been pre-tested [10], rather than validated measures of potential benefits to mentees. Mentoring schemes that focus on women and under-represented researchers have been introduced, but similarly have not been evaluated rigorously despite the potentially important role that they play [11,12]. Such studies [11,12] demonstrate benefits in terms of increased scholarly productivity and academic advancement, suggesting that mentoring may be key to addressing gender and ethnic inequalities. However an understanding of the potential mechanisms by which mentoring may influence and improve career trajectories and professional development is lacking. The existing literature suggests that mentored individuals report increased confidence, and self-esteem compared to their non-mentored counterparts [13], but this has not been evaluated formally to date in schemes which are targeted at women and other underrepresented minority academic faculty [11,14]. There is also little literature concerning pre-mentoring expectations in comparison to what mentees perceive they have achieved and what mentors perceive they have gained from the experience [15].

Therefore we introduced a pilot mentoring scheme for female academics at the Institute of Psychiatry, King's College London, UK. We undertook a comprehensive longitudinal evaluation of our scheme collecting data from both mentee and mentor at baseline, 6 months and 1 year. We aimed to: (i) pilot the mentoring scheme, (ii) evaluate the health-related and attitudinal benefits mentees gained from the experience in terms of self-esteem, self-efficacy, job-related wellbeing, work interference with family and job satisfaction and (iii) compare both mentee and mentor pre-mentoring expectations, to achievements and gains at 6 months and 1 year follow-up. Our study used quantitative measures in an academic setting to ask the research question, what are the mechanisms by which mentoring may support professional development in under-represented groups?

## Method

### Overview

The pilot mentoring scheme for female academics was launched at the Institute of Psychiatry, King's College London, UK, in July 2008 as part of a series of interventions across the University (The Womens' Advancement Initiative). The Institute of Psychiatry is a school of King's College London, with approximately 1,000 members of staff, 42% of whom are female academics, many of whom have clinical as well as academic roles. In 2007, when the scheme was set up, only 26% of professors were women, compared to 61% of lecturers (ratio female: male professors 1: 2.8; female: male lecturers 1: 0.6). The scheme was developed during a consultation process to establish best practice which involved a series of interviews with key stakeholders/opinion leaders who were involved in the setting up and delivery of formal mentoring schemes in the UK and USA and/or positive gender action schemes (further details available from the authors upon request).

### Study participants

Mentees self-selected to be part of the scheme, from a potential participant pool comprising all female academic staff of senior lecturer level or below who had a contract with the Institute of Psychiatry of at least 1 year. Potential mentees were identified from staff lists and were sent information packs and invitations for 'taster lunches'.

Mentors (both male and female to maximise the number of senior individuals) were recruited simultaneously, by invitation (on the basis of recommendations from junior colleagues), by self-sign up after publicity of the scheme, or by specific nomination by an individual mentee at the point of recruitment into the scheme. Strict selection criteria were not applied as the most important factor was motivation to participate in this pilot study.

Mentees were matched with a mentor who they had either nominated, by browsing 'pen portraits' of available mentors, or were matched in a 'clinic' run by the scheme organiser (ACI) - in which 17 potential mentees were interviewed and matched to appropriate mentors according to their seniority level and requirements from mentoring.

All potential mentors were offered 1 1/2 days of training (ACI and Professor David Clutterbuck; http://www.gptrainingconsultants.com). This consisted of an initial training session in key skills of developmental mentoring [16] in advance of their first mentoring meeting, and then 2 further half day sessions that helped mentors develop skills and troubleshoot any problems encountered during mentoring. Mentors also had access to ongoing supervision as needed (ACI).

Mentees received training at an introductory lunch and 1 1/2 hour session explaining the basics of what the scheme involved and how to join the scheme, as well as written information about the purpose of the scheme. Mentoring pairs were advised to attempt to meet between 4-12 times during the year.

### Data collection

Mentors and mentees completed an online survey at the beginning of their mentoring relationships. Further surveys followed at 6 months and 1 year. As well as completing the measures (described below and chosen because they were amenable to short-term change) participants were asked to feedback in free text on the process of the mentoring scheme itself (e.g. registration, matching, training, how many meetings they had had, what was discussed and any perceived benefits or gains of mentoring).

### Quantitative measures

#### Job satisfaction

assessed using 6 items from the satisfaction scale developed by Warr, Cook and Wall [17]. A 5-point Likert scale was used to assess satisfaction with aspects that are intrinsic to a job, including chance of promotion and career support available. The items were summed to give a total score ranging from 6-30. The internal reliability of the scale was 0.82 at pre-mentoring, 0.78 at 6 months and 0.82 at 1 year as measured by Cronbach's alpha.

#### Job-related well-being

assessed using the anxiety-contentment and depression-enthusiasm scales developed by Warr [18,19]. Mentees were asked to indicate how much of the time, in the past few weeks, their job had made them feel a variety of reactions, on a 5-point scale. The responses were averaged across the items so the score ranged from 1 to 5. Cronbach's alpha for the 6-item anxiety-contentment scale was 0.87, 0.88 and 0.80 at pre-mentoring, 6 months and 1 year respectively, and for the 6-item depression-enthusiasm scale was 0.84 at pre-mentoring, 0.87 at 6 months and 0.87 at 1 year.

#### Self-esteem

measured with the Rosenberg Self-Esteem Scale [20], which includes ten items, five of which are positively worded and five are negatively worded. Each was rated on a 4-point scale (0-3), with total scores ranging from 0 (lowest self-esteem) to 30 (highest self-esteem). The internal consistencies were 0.84, 0.88 and 0.84 at pre-mentoring, 6 months and 1 years respectively, as assessed by coefficient alpha.

#### Self-efficacy

assessed using three items developed by Schwarzer and Jerusalem [21]. Responses were on a 5-point scale with scores ranging from 3 to 15. Cronbach's alpha was 0.72 at pre-mentoring, 0.71 at 6 months and 0.51 at 1 year.

#### Work-family conflict

*a*ssessed using four items developed by Kopelman, Greenhaus and Connolly [22]. Responses were on a 5-point scale with scores ranging from 4 to 20. Cronbach's alpha was 0.84 at pre-mentoring, 0.89 at 6 months and 0.90 at 1 year.

Refer to Additional file 1 for the questions used from the various instruments.

### Qualitative measures

A scoping survey, included in the initial information pack for potential mentees and mentors, asked them to identify what they expected to gain or achieve from the process. This was used to inform the themes described as mentee and mentor expectations and achievements/gains (scales available from authors).

### Analysis

#### Quantitative measures

Changes in the scale scores between the pre-mentoring survey and the 6 month survey and the pre-mentoring survey and the 1 year survey were tested using paired t tests. Changes between the 6 month and 1 year surveys were also examined but are not presented (data available from authors upon request).

To evaluate whether mentees who engaged in regular mentoring sessions derived different or greater personal benefits, a sub-analysis of regularly mentored individuals was conducted. 'Regularly mentored' mentees were defined as those who had at least 3 meetings with their mentor in the first 6 months of the scheme and at least 2 meetings in the latter 6 months.

The expectations which the highest numbers of mentees/mentors endorsed were compared with the achievements perceived at 6 months and 1 year using McNemar's test for matched cohort data [23].

#### Qualitative measures

Two independent raters (RD, SLH) applied qualitative principles of content analysis and charting [24] to interpret the free text data regarding the benefits/gains of mentoring. Responses were transcribed onto a word processor. Initially, opinions raised in the transcripts were highlighted and then, using a cut-and-paste technique and Excel spreadsheet, extracted under headings or themes.

### Ethics

The Psychiatry, Nursing & Midwifery Research Ethics Subcommittee of King's College London approved the research protocol (2008). Demographic information on participants in the scheme was not collected to ensure confidentiality, so some analyses were limited by this.

## Results

**Figure 1:**
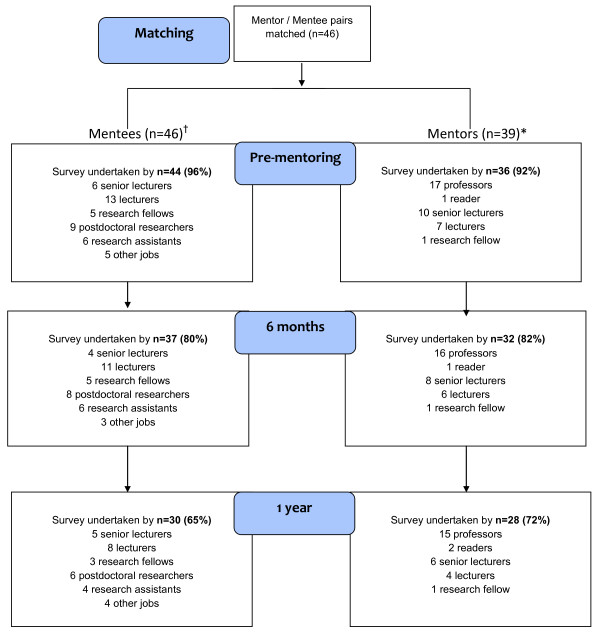
**Flow diagram of online survey completion by Mentees and Mentors**. ^†^n = 37 Mentees undertook both pre-mentoring & 6 month online surveys; n = 29 Mentees undertook both pre-mentoring & 1 year surveys *7 Mentors had 2 mentees each; n = 28 Mentors undertook both pre-mentoring & 6 month online surveys; n = 24 Mentors undertook both pre-mentoring & 1 year surveys.

A total of 46 mentoring pairs were formed; 7 mentors had 2 mentees each (Figure 1).

The 44 (96%) mentees who completed the pre-mentoring survey ranged from research assistant to senior lecturer and five held other roles (PhD student, research nurse, clinical research worker, project coordinator and academic psychiatry specialist registrar). There were 39 mentors. Of the 36 (92%) mentors who completed the pre-mentoring survey, 17 (47%) were professors and the rest ranged from research fellow to reader.

The mentee response rate at 6 months was 80% (37/46). Overall, 30 of the 46 mentees (65%) undertook the survey at 1 year, 6 non-responders were on maternity leave and 3 had left employment at the Institute of Psychiatry. Response rate from mentors was 82% (32/39) at 6 months and 72% (28/39) at 1 year (Figure 1).

### Mentees

There was no improvement in job satisfaction scores at either 6 months or 1 year, but at 1 year there was a significant increase in job-related anxiety-contentment (p = 0.004), although not in job-related depression-enthusiasm (Table 1). The Rosenberg self-esteem score increased by a mean of 2.22 points at 6 months compared to the pre-mentoring survey (p = 0.002) and by a mean of 2.62 points at 1 year (p < 0.001). Schwarzer's self-efficacy score also showed a significant increase at 6 months (p = 0.02) and at 1 year (p = 0.001). Although the 'work interference with family' score showed no difference at 6 months, by 1 year there appeared to be a reduction in work-family conflict (p = 0.04).

**Table 1:** Comparison of mentee scores between the pre-mentoring survey and 6 month and 1 year surveys

	Completed pre-mentoring & 6 month surveys (n = 33)				*Completed pre-mentoring & 1 year surveys (n = 27)*			
	**Pre-mentoring** **mean (s.d.)**	**6 month** **mean (s.d.)**	**Difference in mean at 6 month** **v. Pre-mentoring** **mean (95% CI)**, **p-value**	**No. mentees with improved score at** **6 months**	**Pre-mentoring** **mean** **(s.d.)**	**1 year** **mean (s.d.)**	**Difference in mean** **at 1 year** **v. Pre-mentoring mean (95% CI)**, **p-value**	***No. mentees with improved score at*** ***1 year***

**Job Satisfaction Scale** **Score**	19.50 (5.14)	18.53 (5.25)	0.97 (-0.20 to 2.14), p = 0.10	10	18.78 (4.76)	17.81 (5.23)	0.96 (-0.57 to 2.49), p = 0.21	*9*

**Job-related Anxiety-** **Contentment Score**	2.80 (0.82)	2.87 (0.79)	0.07 (-0.20 to 0.33), p = 0.62	16	2.68 (0.70)	3.07 (0.64)	0.40 (0.14 to 0.65), p = 0.004*	*20*

**Job-related Depression-Enthusiasm Score**	3.54 (0.67)	3.56 (0.83)	0.02 (-0.19 to 0.22), p = 0.88	12	3.48 (0.69)	3.59 (0.61)	0.11 (-0.16 to 0.38), p = 0.40	*16*

**Rosenberg Self-esteem Scale Score**	19.66 (4.55)	21.88 (4.75)	2.22 (0.87 to 3.57), p = 0.002*	19	20.62 (3.82)	23.20 (3.89)	2.62 (1.36 to 3.87), p < 0.001*	*20*

**Schwarzer's Self-efficacy** **Scale Score**	10.45 (1.87)	11.36 (1.50)	0.91 (0.18 to 1.64), p = 0.02*	17	10.48 (1.74)	11.56 (1.42)	1.07 (0.46 to 1.68), p = 0.001*	*14*

**Work Interference with Family Score**	13.52 (3.65)	13.30 (3.91)	-0.21 (-1.44 to 1.02), p = 0.73	13	14.41 (3.60)	12.89 (3.92)	-1.52 (-0.09 to -2.95), p = 0.04*	*14*

s.d. - standard deviation; CI - confidence interval; *- p < 0.05

We repeated these analyses for the 21 'regularly mentored' mentees who completed scales at pre-mentoring and 6 months, the 20 'regularly mentored' mentees who completed scales at pre-mentoring and 1 year and the 13 'regularly mentored' mentees who completed scales at all three time points (pre-mentoring, 6 months and 1 year). Results from the sub-analysis of 'regularly mentored' mentees was consistent with the analysis of the complete dataset (results not shown, data available from the authors).

The five mentee expectations which were most heavily endorsed were career progression (39/44; 89%), increased confidence (38/44; 87%), development of networking skills (33/44; 75%), better time-management (29/44; 66%) and better work-life balance (28/44; 64%). These top five expectations were also the highest perceived achievements at the two later time points (Table 2). In all cases, prior expectations were higher than perceived achievements at 6 months and 1 year. The Expectation to achievement ratios were particularly high with regard to career progression, development of networking skill and expectation of a better work-life balance (Table 2). However, three key themes did emerge as perceived benefits of mentoring to mentees: (i) confidence and assertiveness, (ii) support and encouragement and (iii) space to reflect on careers and goals.

**Table 2:** Comparison of mentees' pre-mentoring expectations versus achievements at 6 months and 1 year

Mentee expectations	Expected^#^ (pre-mentoring) (%)	Achieved^#^ (6 months) (%)	Ratio of Expectation: Achievement (6 months)	Expected^#^ (pre-mentoring) (%)	Achieved^#^ (1 year) (%)	*Ratio of Expectation: Achievement* *(1 year)*
**Career progression**	30/34 (88)	20/34 (59)	1.50 (p = 0.03*)	23/25 (92)	10/25 (40)	2.30 (p < 0.001*)

**Increased confidence**	29/31 (94)	23/31 (74)	1.26 (p = 0.07)	23/25 (92)	19/25 (76)	*1.21 (p = 0.22)*

**Development of networking skills**	27/34 (79)	17/34 (50)	1.59 (p = 0.006*)	19/25 (76)	11/25 (44)	1.73 (p = 0.04*)

**Better time-management**	23/34 (68)	19/34 (56)	1.21 (p = 0.34)	18/25 (72)	12/25 (48)	*1.50 (p = 0.15)*

**Better work-life balance**	23/32 (72)	12/32 (38)	1.92 (p = 0.01*)	20/24 (83)	12/24 (50)	1.67 (p = 0.04*)

^#^Denominators for each expectation/achievement vary, because not all mentees answered each item; *- p < 0.05

### Mentee perceptions of the benefits of mentoring

(i) Confidence and assertiveness

• the mentoring program "... gave me confidence to apply for a [promotion]"

• "the mentoring program has made me more assertive to ask for more in the workplace"

(ii) Support and encouragement

• ".. .encouragement to do some new and challenging things for the benefit of my career"

• "invaluable to have someone outside my section, but within my department who has some insight into the frustrations of being mid-career and is able to give some useful support"

(iii) Space to reflect on careers and goals

• "... support, encouragement, a space for thinking and talking about work issues and my career and thinking about what I want, practical strategies and information"

### Mentors

For mentors, the five highest expectations were of satisfaction in seeing people progress (25/36; 69%), seeing junior staff develop and grow (19/36; 53%), helping to solve problems (18/36; 50%), helping women advance their careers (18/36; 50%) and helping to remove obstacles to their careers (13/36; 36%). However, when measuring perceived gains at 6 months and 1 year, the proportion of mentors who endorsed the statement: 'a good relationship with my mentee', was ranked as one of the highest gains (22/32; 69% of respondents at 6 months), although it was not a high expectation at outset (10/36; 28%). In most cases, gains and expectations were similar but there were sometimes gains at 6 months and 1 year that had not been expected at the pre-mentoring stage. This was especially true for having a good relationship with the mentee and for helping to solve problems (Table 3). Four key themes emerged as perceived gains: (i) being able to give something back, (ii) to see the Institute of Psychiatry from a different perspective, (iii) providing support and seeing mentee develop and (iv) reflection on own career and skills.

**Table 3:** Comparison of mentors' gains at 6 months and 1 year versus pre-mentoring expectations

Mentor expectations	Expected^#^ (pre-mentoring) (%)	Gained^#^ (6 months) (%)	Ratio of Gain: Expectation (6 months)	Expected^¶^ (pre-mentoring) (%)	Gained^¶^ (1 year) (%)	*Ratio of Gain: Expectation* *(1 year)*
**Satisfaction in seeing people progress**	18/28 (64)	20/28 (71)	1.11 (p = 0.79)	16/24 (67)	18/24 (75)	*1.13 (p = 0.77)*

**To see junior staff develop and grow**	16/28 (57)	14/28 (50)	0.88 (p = 0.69)	14/24 (58)	13/24 (54)	*0.93 (p = 1.00)*

**Helping to solve problems**	13/28 (46)	18/36 (64)	1.38 (p = 0.30)	10/24 (42)	18/24 (75)	1.80 (p = 0.04*)

**Helping women advance their careers**	15/28 (54)	17/28 (61)	1.13 (p = 0.73)	12/24 (50)	11/24 (46)	*0.92 (p = 1.00)*

**Helping to remove obstacles to career**	9/28 (32)	8/28 (29)	0.89 (p = 1.00)	7/24 (29)	6/24 (25)	*0.86 (p = 1.00)*

**A good relationship with my mentee**	8/28 (29)	20/28 (71)	2.50 (p = 0.002*)	8/24 (33)	16/24 (67)	2.00 (p = 0.04*)

^#^28 mentors completed all expectation and gain items at both the prementoring and 6 month survey

^¶^24 mentors completed all expectation and gain items at both the prementoring and 1 year survey *- p < 0.05

### Mentor perceptions of the benefits of mentoring

(i) Being able to give something back

• "... to pass on some of the insights that I have gained from my experiences at the Institute of Psychiatry to the next generation"

(ii) To see the Institute of Psychiatry from a different perspective

• "... learning about a different working culture and different challenges in another Institute of Psychiatry department (and being grateful that these factors don't seem so bad within my own!)"

(iii) Providing support and seeing mentee develop

• "... knowing that my mentee is now feeling better about the issues she came with and is more optimistic about her career progression than when I first met her"

(iv) Reflection on own career and skills

• "I have been very impressed with my mentee's resilience in the face of some very major issues in her life. This has made me reflect on some of my own ways of dealing with challenges"

## Discussion

Self-esteem, self-efficacy and job-related well-being (anxiety-contentment) improved significantly with mentoring. Work-family conflict had diminished at 1 year follow-up. Improved confidence and assertiveness, receipt of support and encouragement and the space to reflect on careers and goals emerged as important benefits. However neither the mentee job satisfaction scores increased nor was the pre-mentoring expectation of 'career progression' achieved by 1 year. For mentors the perceived gains at 6 months and 1 year often exceeded pre-mentoring expectations; mentoring yielded some unexpected rewards.

### Self-esteem

Our results regarding a significant improvement in self-esteem at 6 months and 1 year are consistent with Ragins and colleagues examination of the mentee perspective of mentoring in a management setting, which found that mentored individuals had significantly higher levels of organizational based self-esteem than their non-mentored counterparts [13]. Although levels of self-esteem have been measured (also using Rosenberg's Scale) in mentoring programmes for college students [25], to our knowledge self-esteem has not been studied within an academic mentoring context. It seems likely that such improvements are related longer term to career progression, but the short time scale of our pilot meant that we were unable to demonstrate this.

### Self-efficacy

Self-efficacy is the belief that one has the capabilities to execute the courses of actions required to manage a prospective situation and is the central construct in Bandura's social cognitive theory [26]. It is thought that perceptions of academic and relational self efficacy result in the perseverance and resilience required to overcome career obstacles [27] and as such, improvement in self efficacy is a key outcome measure most clearly allied with the aims of mentoring. In a qualitative study of 15 women who selected and excelled at academic careers in mathematics, science and technology, good mentors were identified as those who had confidence in their mentees' abilities and in doing so, enhanced their mentee's self efficacy [27]. The apparent increase in self-efficacy over the course of our pilot mentoring study is an interesting confirmatory finding of the importance of mentoring in improving this construct for female academics.

Both women's global self-esteem (which captures self-assessed overall worth) and task-specific self-efficacy (which captures the self-assessed ability to perform a certain task) have been shown to be predictors of self-limiting behaviour in certain leadership tasks [28]. In a study involving female psychology and business students at University, Dickerson and Kay found that women with high self-efficacy were more likely to attempt leadership tasks and express interest in performing them, whereas those with lower self-efficacy were more likely to choose a subordinate task and self-select out of a leadership role. The authors suggest that mentoring may be helpful in providing timely, accurate and specific feedback to women and help them to plan strategies to improve their performance [28]. Our study also suggests mentoring could improve self-efficacy for future tasks.

### Job-related well-being

We noted that there was no difference between job-related depression-enthusiasm at 6 months or 1 year compared to pre-mentoring, whereas there was an improvement in the anxiety-contentment scale. Mentoring may be a factor in improving contentment at work, as suggested by a review concerning 'authentic happiness' in dentists, as a result of mentoring within professional development programmes [29]. There may be specific aspects of job-related mood states depending on the stresses associated with different professions, and in the context of the Institute of Psychiatry, this appeared to be anxiety-contentment.

### Work interference with family

The work interference with family scale was used to understand whether mentors play a role in influencing mentees' perceptions of the work-family interface. It appeared by 1 year that there was some overall reduction in the level of perceived conflict. In a field study of 502 male and female graduates from American business management courses, who were either involved in a committed relationship or had children living at home, it was found that individuals with mentors reported significantly less work-family conflict than those without mentors [30]. However this was particularly in the dimension of family interference with work, rather than work interference with family, suggesting that both directions of work-family conflict should be considered in future studies. Women more commonly report work-life conflict due to their multiple roles and some evidence indicates that the negative effects of work-family conflict may be greater for women [31]. This would suggest that if conflict could be reduced by interventions including strong mentors, more female academics could be retained and progress in their careers [32].

### Job satisfaction

Our inability to demonstrate an improvement in job satisfaction was unsurprising given that at the time the mentoring scheme was introduced and evaluated at the Institute of Psychiatry, it was in the context of considerable institutional and national economic uncertainty with a local freeze on posts, and a 'Sustainability Review' at the Institution which involved redundancies. The fact that despite this, there were improvements in many of our outcome measures suggests that at an individual level, mentoring was still of benefit for mentees.

### Mentee expectations

The high levels of mentee expectations which we found at the pre-mentoring stage have also been reported in some cases studied by Wildman et al [33] of experienced teachers mentoring beginning teachers in schools. More initial training of mentees could instil more realistic expectations of the mentoring process and be helpful in aligning expectations with more realistic outcomes.

### Mentor expectations

It is noteworthy that in many areas, the mentors appeared to gain more from their experiences than they expected. Clutterbuck notes that powerful often unpredicted benefits exist for mentors and these often prompt reassessment of their own views and leadership style [16]. A modified version of this theme - 'reflection on own career and skills' - emerged from our qualitative analysis. This new evidence, if replicated, would be a reason to include the experience of being a mentor as a valuable part of continuous professional development for senior staff in academic roles, in the future.

### Limitations

Our study has several limitations. First, it was an uncontrolled pilot study, aimed at feasibility and establishing any effects. The lack of any control group means that our results need to be seen as indicative only. Second, the limited numbers of mentees and mentors in this pilot study reduces the power of any statistical analysis and to protect staff anonymity, mentee demographic data (e.g. age, ethnicity, number of children) were not collected. Third, there was some missing data at each time point which means that the paired t tests and matched McNemar's tests were based only on complete data. Fourth, all the mentors and mentees were self-selected and their interest in mentorship may have skewed the results towards a more favourable estimate of the personal gains and achievements from mentoring, and of higher and more unrealistic expectations. Fifth, the spectrum of type and rank of academics involved in this small study was broad, and the results are not representative of one particular occupational group or demographic, although all staff worked in a specialist academic mental health institution. Sixth, objective outcomes of career progression (such as publishing research papers or obtaining grants) were not included because of the time scale.

### Future work

This was a feasibility pilot study of mentoring. To determine the true effect of mentoring in an institution a randomised controlled trial would be an important next step.

In any trial which builds on this pilot work it will be important to also track objective outcome measures such as mean number of publications, change in H-index, number of research grants awarded and promotion within academia, as indicated by Sambunjak et al [9] in their systematic review. It will also be necessary to follow-up participants over a longer time period of as much as 5-10 years, as 'career progression' outcomes are unlikely to be apparent at a 6 month or 1 year follow-up. Self-perceptions (self-esteem and self-efficacy) warrant further study using more comprehensive measures in future research. Also the themes which emerged as important perceived benefits in the qualitative analysis (i.e. self-confidence and assertiveness) could be analysed in a quantitative manner using well validated and reliable scales to see if these qualities improved objectively. In academic psychiatry, it has been suggested that choosing senior female mentors for junior women might encourage female trainees to consider this specialty [34]. In future work it will be pertinent to analyse whether the gender of the mentor affects mentees' perceptions of mentoring and personal gains. We also intend to examine the quality of the mentor-mentee relationship and specific mentor functions as perceived by mentees.

## Conclusion

The potential benefits of academic mentoring for women are important. This pilot study has indicated that mentoring could contribute to women's personal and professional development. The study was also able to begin to demonstrate the mechanisms involved. Rigorous evaluation of mentoring as an intervention would be the next step for institutions which want to help retain and develop the careers of their academic staff, particularly their women academics.

## Competing interests

The authors declare that they have no competing interests.

## Authors' contributions

RD reviewed the literature, undertook the statistical analyses, interpreted the data and drafted the manuscript which was critically revised by the other authors. SLH participated in the design, collected the data and was responsible for the coordination of the study. EK helped conceive and design the study, protocol and interpret the data. DG was involved in the design of the study, and the choice of measures, and commented on the manuscript. NTF and ACI conceived of and designed the study, wrote the protocol and participated in interpretation of the data. NTF assisted with the statistical analysis. All authors read and approved the final manuscript.

## Pre-publication history

The pre-publication history for this paper can be accessed here:

http://www.biomedcentral.com/1472-6920/11/13/prepub

## Supplementary Material

Additional file 1 **Appendix**. Questions used as quantitative measures. Click here for file
