# Supplementary material for: One year outcomes of a mentoring scheme for female academics: a pilot study at the Institute of Psychiatry, King's College London
Source: BMC Med Educ. 2011 Apr 7;11:13. doi: 10.1186/1472-6920-11-13 (PMC3094330; doi:10.1186/1472-6920-11-13)
Supplement: Additional file 1 — Appendix. Questions used as quantitative measures. [file 1472-6920-11-13-S1.DOC]

**Appendix**

**Job Satisfaction Scale - 6 items adapted from Warr, Cook and Wall’s scale (1979)**

Please indicate how satisfied or dissatisfied you feel with each of the following statements:

1. Your current job

2. Working at the Institute of Psychiatry

3. Your career at the Institute of Psychiatry

4. The level of pay you currently receive

5. Promotion prospects at the Institute of Psychiatry

6. Career support at the Institute of Psychiatry

Responses were given on a 5-point Likert scale 1=very dissatisfied, 2=somewhat dissatisfied, 3=neutral, 4=somewhat satisfied, 5=very satisfied

**Job-related Well-being Scale – Warr (1990)**

Thinking of the past few weeks, how much of the time has your job made you feel each of the following:

1. Tense
2. Miserable
3. Depressed
4. Optimistic
5. Calm
6. Relaxed
7. Worried
8. Enthusiastic
9. Uneasy
10. Contented
11. Gloomy
12. Cheerful

Responses were given on a 5-point Likert scale 1=Never, 2=Occasionally, 3=Some of the time, 4=Most of the time, 5=All of the time

The Anxiety-Contentment scale consists of items 1,5,6,7,9 and 10; and the Depression-Enthusiasm scale of items 2,3,4,8,11 and 12.

Negative items (1,2,3,7,9 and 11) were reverse scored and responses were averaged across the items to provide the scale score.

**Rosenberg’s Self-Esteem Scale (1965)**

1. I feel that I am a person of worth, at least on an equal plane with others.

2. I feel that I have a number of good qualities.

3. All in all, I am inclined to feel that I am a failure.

4. I am able to do things as well as most other people.

5. I feel I do not have much to be proud of.

6. I take a positive attitude toward myself.

7. On the whole, I am satisfied with myself.

8. I wish I could have more respect for myself.

9. I certainly feel useless at times.

10. At times I think I am no good at all.

Scoring: for items 1, 2, 4, 6, and 7:

Strongly agree = 3

Agree = 2

Disagree = 1

Strongly disagree = 0

For items 3, 5, 8, 9, and 10 (which are reversed in valence):

Strongly agree = 0

Agree = 1

Disagree = 2

Strongly disagree = 3

The scale ranges from 0-30. Scores between 15 and 25 are within normal range. Scores below 15 suggest low self-esteem.

**Occupational self-efficacy scale – 3 items adapted from Schwarzer’s Self-efficacy Scale (1995)**

Please indicate the extent to which you agree or disagree with the following statements:

1. I can remain calm when facing difficulties in my job because I can rely on my abilities

2. When I am confronted with a problem in my job, I can usually find several solutions

3. Whatever comes my way in my job, I can usually handle it

Responses were given on a 5-point Likert scale 1=Strongly disagree, 2=Disagree, 3=Neither agree nor disagree, 4=Agree, 5=Strongly agree.

**Work Interference with Family (WIF) Scale – 4 items adapted from Kopelman, Greenhaus and Connoly (1983)**

Please indicate the extent to which you agree or disagree with the following statements:

1. After work, I am too tired to do some of the things I’d like to do

2. At work, I have so much to do that it takes away from my personal interests

3. My family / friends dislike how often I am preoccupied with my work when I’m not working

4. My work takes up time that I’d like to spend doing other things

Responses were given on a 5-point Likert scale 1=Strongly disagree, 2=Disagree, 3=Neither agree nor disagree, 4=Agree, 5=Strongly agree.
